# Development and Gelation Mechanism of Ultra-High-Temperature-Resistant Polymer Gel

**DOI:** 10.3390/gels9090726

**Published:** 2023-09-07

**Authors:** Zhenfeng Ma, Mingwei Zhao, Ziteng Yang, Xiangyu Wang, Caili Dai

**Affiliations:** 1 National Key Laboratory of Deep Oil and Gas, China University of Petroleum (East China), Qingdao 266580, China; mzf17852157017@163.com (Z.M.); 19863704412@163.com (Z.Y.); wangxy7918@163.com (X.W.); daicl@upc.edu.cn (C.D.); 2 Shandong Key Laboratory of Oilfield Chemistry, China University of Petroleum (East China), Qingdao 266580, China

**Keywords:** polymer gel, ultra-high-temperature-resistant, rheology property, gelation mechanism

## Abstract

To expand the applicability of gel fracturing fluids in ultra-high-temperature reservoirs, a temperature-resistant polymer was synthesized using the solution polymerization method. Subsequently, an ultra-high-temperature-resistant polymer gel was formulated by incorporating an organic zirconium crosslinking agent. A comprehensive investigation was carried out to systematically study and evaluate the steady shear property, dynamic viscoelasticity, and temperature and shear resistance performance, as well as the core damage characteristics of the polymer gel. The obtained results demonstrate that the viscosity remained at 147 mPa·s at a temperature of 200 °C with a shear rate of 170 s^−1^. Compared with the significant 30.9% average core damage rate observed in the guanidine gum fracturing fluid, the core damage attributed to the polymer gel was substantially mitigated, measuring only 16.6%. Finally, the gelation mechanism of the polymer gel was scrutinized in conjunction with microscopic morphology analysis. We expect that this study will not only contribute to the effective development of deep and ultradeep oil and gas reservoirs but also furnish a theoretical foundation for practical field applications.

## 1. Introduction

In recent years, the exploration of unconventional oil and gas resources has garnered significant attention due to the diminishing accessibility of conventional reserves [1,2]. The potential of deep and ultradeep reservoirs, surpassing depths of 4500 m, to yield substantial oil and gas resources has become increasingly recognized. However, these reservoirs are confronted with formidable global challenges. These challenges encompass the imperative for drilling remarkably deep wells, elevated prerequisites for mechanical equipment, intricacies in drilling methodologies, judicious selection of operational fluids, and the reservoir environments characterized by elevated pressures, high temperatures, and pronounced salinity levels [3,4,5,6]. Furthermore, these reservoirs exhibit distinctive physical attributes, typified by predominantly nano–micro-sized pores and throats, coupled with considerable reservoir heterogeneity, which curtails oil production. Conventional water-driven extraction methods encounter limitations in these complex reservoirs, leading to suboptimal oil recovery, escalated operational expenses, and significant resource wastage [7]. In response to these issues, fracturing technology has emerged as a paramount approach for exploiting unconventional oil and gas resources. The fracturing process involves injecting high-pressure working fluids into subsurface formations, inducing a network of microscopic fractures within the rock structure [8,9]. This fracture network establishes connectivity within the reservoir, amplifying fluid flow capacity and ultimately augmenting production. Given the extraction of oil and gas resources from greater depths and diverse physical conditions, the efficacy of fracturing techniques as sumes pivotal importance.

Fracturing operations are profoundly impacted by the efficacy of fracture length and inflow capacity, both intricately linked to the attributes and functional requisites of fracturing fluids [10,11,12]. The most widely used water-based fracturing fluids encompass various types, including slickwater fracturing fluids [13], clean fracturing fluids [14], and gel fracturing fluids [15]. While slickwater fracturing fluids excel in drag reduction, their temperature resistance and sand-carrying capacity might be restricted [16]. Clean fracturing fluids, devoid of residues after gel breaking and causing minimal reservoir damage, face challenges due to their elevated costs and limited temperature endurance [17]. In contrast, gel fracturing fluids showcase notable capabilities in temperature and shear resistance, along with effective sand-carrying properties [18,19]. These traits align well with the specific requirements of deep and ultradeep reservoirs, thus positioning gel fracturing fluids as a highly promising solution for practical implementation.

Gel fracturing fluids are primarily composed of gels formed through the combination of thickeners and crosslinking agents [20,21,22]. These formulations also integrate suitable additives, such as gel breakers and weighting agents, to meet specific performance criteria. Thickeners, often water-soluble polymers, play a crucial role by augmenting the viscosity and energy transfer capability of the base fluid due to their intricate interlinked structure [23]. Current research efforts concerning thickeners tailored for ultra-high-temperature-resistant gels predominantly revolve around acrylamide polymers [24,25,26,27]. These linear polymers feature carbon–carbon bonds as their primary chains, often incorporating various functional monomers and groups as required. The high energy associated with carbon–carbon bonding and its exceptional thermal stability contribute to effective viscosity enhancement [28]. Further enhancements in overall temperature and shear resistance can be achieved by introducing monomers with benzene rings, polycyclic rings, or substantial side groups [29,30,31]. These modifications induce spatial steric effects within the polymer, thereby boosting its temperature resistance. The crosslinking agent plays a pivotal role in gel formation by interacting with the crosslinking structural units present in the polymer molecules. Active groups, such as amide and carboxyl, within the polymer molecular chains facilitate this interaction [32,33]. The crosslinking process results in the formation of three distinct types of chemical bonds, including ligand, covalent, and ionic bonds [34]. This intricate bonding mechanism ultimately imparts the gel with remarkable temperature resistance. Zhao et al. [35] developed an innovative acid gel with excellent fundamental properties and elevated viscosity, suitable for acid fracturing operations in reservoirs with temperatures up to 120 °C. Kamal et al. [36] introduced a novel polymeric gel system capable of enduring formation temperatures reaching 150 °C while maintaining stability for approximately 6 h under high shearing rates (511 s^−1^). Nevertheless, the existing gels may not fully satisfy the temperature demands of ultradeep reservoirs that can reach 200 °C due to their limited temperature resistance. Hence, there exists a pivotal need to synthesize a polymer with outstanding ultra-high-temperature resistance and develop a corresponding polymer gel, both of which hold crucial significance for the successful exploitation of deep and ultradeep oil and gas reservoirs.

In this work, a temperature-resistant polymer was synthesized utilizing the solution polymerization method and subjected to thorough characterization. An ultra-high-temperature-resistant (200 °C) polymer gel was formulated by employing this polymer as the thickener and incorporating an organic zirconium crosslinking agent. The steady shear property, dynamic viscoelasticity, and temperature and shear resistance performance of the ultra-high-temperature-resistant polymer gel were investigated under optimized concentrations of the thickener and crosslinking agent. Additionally, the core damage property was meticulously assessed. Lastly, the gelation mechanism of the polymer gel was explored, accompanied by microscopic morphology analysis. It is expected to yield crucial insights for the research and development of ultra-high-temperature-resistant polymer gel fracturing fluids.

## 2. Results and Discussion

### 2.1. Structure Characterization of the Synthesized Polymer

**Figure 1:**
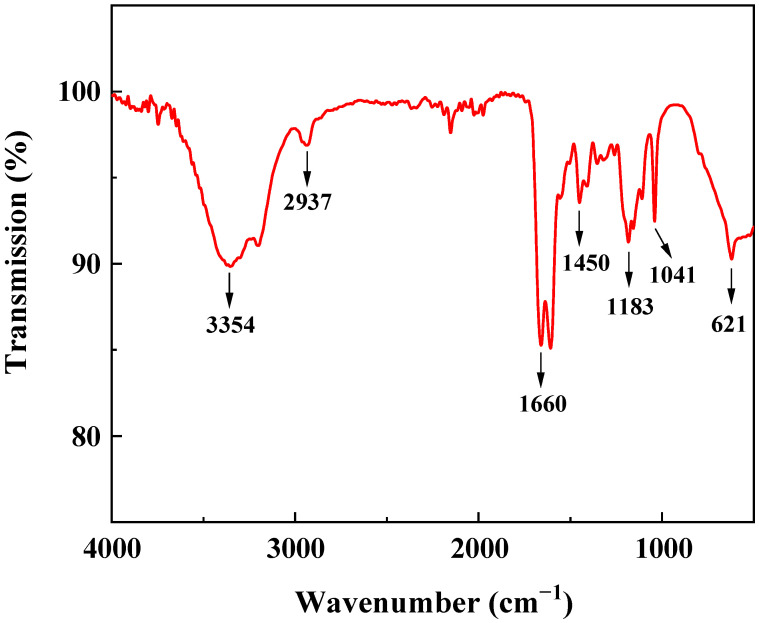
FTIR spectrum of polymer.

Fourier-transform infrared spectroscopy (FTIR) stands as a pivotal technique for characterizing the synthesized polymer [37,38,39]. Figure 1 depicts the FTIR spectra of the polymer, with annotated wavenumbers corresponding to discernible peaks that facilitate the discernment of specific functional groups. The peak observed at 3354 cm^−1^ is indicative of the bending and contraction vibration of –NH_2_ in AM, while the peak at 2937 cm^−1^ originates from the contraction vibration of –CH_3_ in AMPS. The peak situated at 1660 cm^−1^ can be attributed to the contraction vibration of –C=O, and a noticeable shift to 1450 cm^−1^ signifies the presence of –CH_2_. Furthermore, the peaks at 1183 cm^−1^ and 1041 cm^−1^ can be ascribed to the –SO_3_ functional group in AMPS, and a discernible shift to 621 cm^−1^ indicates the existence of C–S bonding. The comprehensive scrutiny of the FTIR spectra furnishes validation of the synthesized polymer.

Thermogravimetric analysis (TGA) techniques are extensively used to investigate the physical properties of samples under varying temperatures, providing valuable insights into the physical properties and thermal stability of materials [40,41,42]. The orange curve delineates the variation in polymer mass percentage with changing experimental temperature, while the left vertical axis quantifies the mass loss. Simultaneously, the green curve corresponds to the thermogravimetric differential curve, portraying the first-order differentiation of points from the thermogravimetric curve in relation to time. This curve characterizes the rate of change in polymer mass percentage, with the right vertical axis illustrating the outcomes of the differentiation of the thermogravimetric curve.

**Figure 2:**
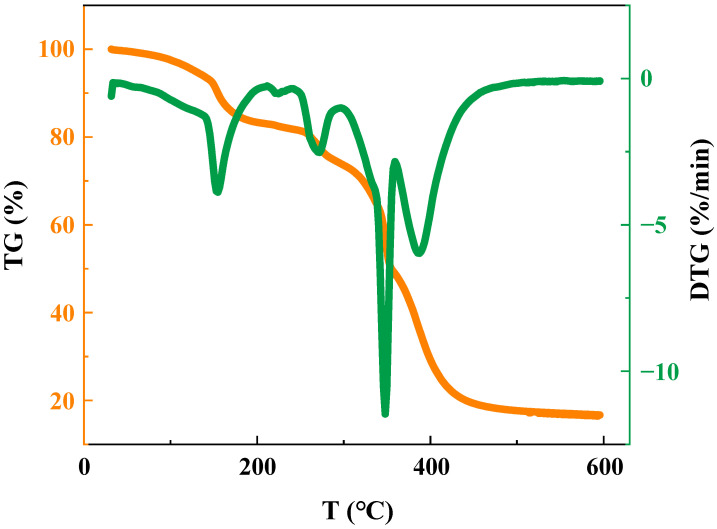
Thermogravimetric curve of polymer.

As illustrated in Figure 2, the mass of polymer experiences gradual reduction with ascending temperature, and the curve delineates three primary stages. The initial stage, spanning from 30 °C to 225 °C, witnesses a progressive mass decline, constituting a cumulative mass loss of 17.1%. This phenomenon is attributed to the evaporation of crystallization water embedded within the polymer molecules upon exposure to heightened temperatures. The ensuing stage unfolds between 259 °C and 280 °C, during which the amide groups in the polymer undergo decomposition under elevated thermal conditions. This leads to a discernible mass reduction, culminating in an aggregate loss of 9.29%. The third stage, spanning from 372 °C to 598 °C, prominently displays a steep downturn in the thermogravimetric profile, accompanied by a significant mass loss of 32.1%. This reduction primarily emanates from the degradation of quaternary ammonium salts alongside a fraction of the sulfonic acid groups. The overarching trajectory delineated by the thermogravimetric curve suggests that the polymer possesses commendable temperature resistance.

### 2.2. Concentration Optimization of Polymer and Crosslinking Agent

**Figure 3:**
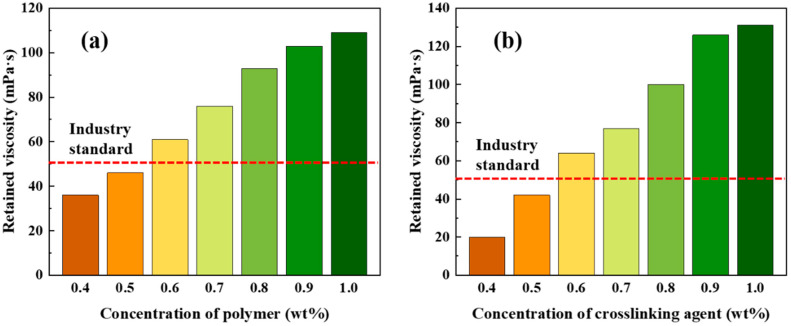
Retained viscosity of gels at (**a**) 0.6 wt% crosslinking agent and different polymer concentrations and (**b**) 0.6 wt% polymer and different crosslinking agent concentrations.

Figure 3 depicts the results of retained viscosity for different gel formulations comprising varying concentrations of polymer and crosslinking agent. Maintaining the crosslinking agent concentration at a constant 0.6 wt%, an escalation in polymer concentration yielded augmented retained viscosity values following the temperature and shear resistance experiments. Subsequently, notable enhancements were observed in temperature and shear resistance performance. Notably, at a polymer concentration of 0.6 wt%, the retained viscosity attained 61 mPa·s at a shear rate of 170 s^−1^, fulfilling the industry standard requirement of 50 mPa·s.

Maintaining a constant polymer concentration of 0.6 wt%, an increase in crosslinking agent concentration led to elevated retained viscosity values after the temperature and shear resistance tests. Notably, at a crosslinking agent concentration of 0.6 wt%, the retained viscosity achieved 64 mPa·s, adhering to the industry standard requirement of 50 mPa·s. Based on these findings, it can be inferred that an optimal combination of 0.6 wt% polymer concentration and 0.6 wt% crosslinking agent concentration yields desirable properties.

### 2.3. Rheological Properties of the Polymer Gel

#### 2.3.1. Steady Shear Property

**Figure 4:**
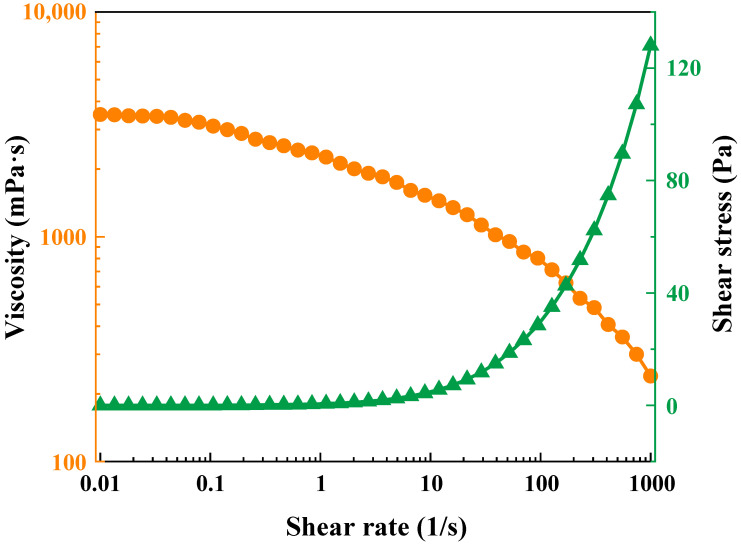
Steady shear curve of the polymer gel.

Figure 4 portrays the alteration in viscosity of the polymer gel across varying shear rates. The polymer gel showcases the attributes of a non-Newtonian fluid. Under low shear rates, the viscosity of gel maintains a relatively consistent profile, with minimal fluctuations. This steadfast viscosity value can be designated as the zero-shear viscosity. With the escalation of shear rates, the gel demonstrates the characteristic behavior typical of non-Newtonian fluids, referred to as the shear-thinning phenomenon. This decline can be attributed to heightened shear rates inducing the rupture of polymer molecular chains, resulting in the disruption of the network structure and subsequent reduction in gel viscosity. Nonetheless, even at elevated shear rates, the gel viscosity remains at 240 mPa·s, signifying the impressive shear resistance inherent to the polymer gel [43,44].

#### 2.3.2. Dynamic Viscoelasticity

**Figure 5:**
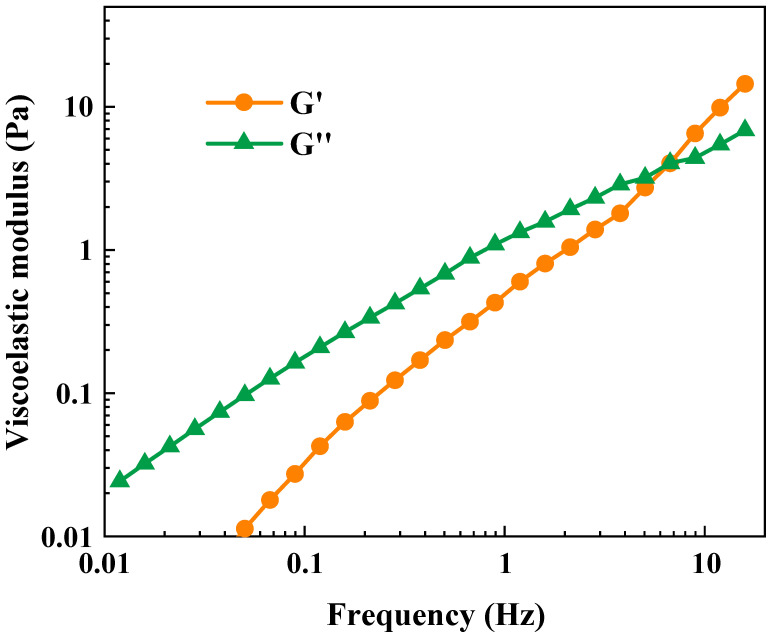
Viscoelasticity curve of the polymer gel.

Figure 5 elucidates the frequency-dependent viscoelastic tendencies of the polymer gel. With increasing frequency, both the storage moduli G′ and the loss moduli G″ exhibit an ascending trajectory, underscoring the relative influence of frequency on the micelle structure during this phase. Remarkably, the storage moduli G′ demonstrates a more rapid escalation compared with the loss moduli G″. At a specific frequency juncture, the G′ and G″ curves intersect. Within the lower frequency spectrum, the G″ surpasses the G′, indicative of the rheological properties of the polymer gel being predominantly governed by its viscous characteristics. Nevertheless, as frequency advances into higher realms, the G′ surpasses the G″, signifying that the rheological behavior of the polymer gel is predominantly influenced by its elastic attributes [45]. This phenomenon can be attributed to the augmented interaction between the hydroxyl bridge complex ions and carboxyl groups existing within the polymer matrix. This behavior underscores the distinctive response of an elastic fluid, thereby providing compelling evidence of its gel-like characteristics.

#### 2.3.3. Temperature and Shear Resistance

**Figure 6:**
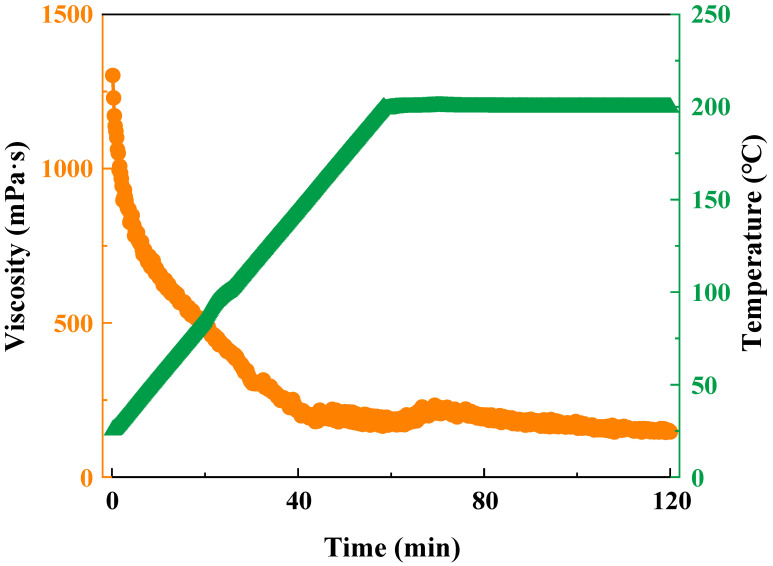
Temperature and shear resistance curve of the polymer gel.

In general, the spatial network structure of the polymer gel is significantly impacted by the formation temperature, leading to a rapid decline in gel viscosity [46,47,48]. Consequently, the temperature and shear resistance of the polymer gel become crucial factors to consider. The results of the tests, as illustrated in Figure 6, depict the behavior of temperature and shear resistance. In the initial 40 min, under a constant shear rate, the network structure of gel is influenced by temperature elevation, resulting in a gradual reduction in gel viscosity. Subsequently, the gel viscosity curve demonstrates fluctuations, indicative of pronounced damage to the molecular chains of the synthesized polymer at elevated temperatures. As the polymer interacts with the zirconium ions in the crosslinking agent to establish a compact network structure, it is concurrently exposed to high-temperature shear damage. Nevertheless, as the network structure begins to grow faster than the rate of destruction, the gel viscosity experiences a slight increase [49]. Eventually, with the complete bonding of zirconium ions to the polymer molecular chains, the gel viscosity starts to decline and stabilize under the influence of shear damage. Remarkably, the gel viscosity remains at 147 mPa·s at 200 °C. Furthermore, even after 60 min of continuous shear at 200 °C, the viscosity remains relatively stable, underscoring the exceptional temperature and shear resistance of the polymer gel.

### 2.4. Core Damage Performance

The introduction of polymer gel into the reservoir can perturb the original dynamic equilibrium of the formation [50,51,52]. This adversarial interaction between the fluid and the formation, leading to diminished permeability of the reservoir, is termed reservoir damage. The magnitude of reservoir damage attributed to polymer gel assumes pivotal significance in determining the efficacy of its application. To assess the extent of reservoir damage caused by the polymer gel in comparison with a conventional guanidine gum fracturing fluid system, experiments were conducted on low-permeability cores possessing similar initial permeability. Table 1 illustrates that the utilization of the prepared polymer gel resulted in a modest average damage rate of 16.6%. In stark contrast, the application of the conventional guanidine gum fracturing fluid system led to a significantly higher average damage rate of 30.9%. The relative standard deviations (RSD) associated with the damage rates for both the polymer gel and the conventional guanidine gum fracturing fluid system were 3.03% and 4.49%, respectively, underlining the reliability of the data. This unequivocally highlights the distinct advantages of the polymer gel in ameliorating reservoir damage, rendering its application more advantageous.

### 2.5. Gelation Mechanism of the Polymer Gel

**Figure 7:**
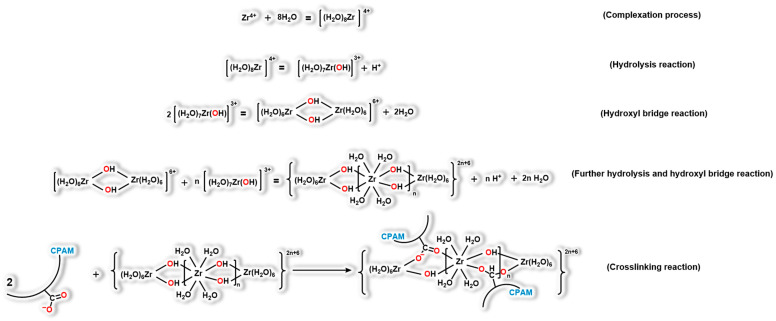
Gelation process of the polymer gel.

In current investigations focused on temperature-resistant polymer gels, the organic zirconium crosslinking agent has emerged as the most widely utilized metal-ion crosslinking agent [53,54,55]. The gelation mechanism involving the polymer and organic zirconium crosslinking agent involves a series of intricate reactions. Specifically, this process can be delineated in Figure 7. Zirconium ions present in the solution initiate the formation of complexes through a complexation process. Subsequent to this, hydrolysis reactions take place, leading to the creation of hydroxyl bridge structures. These hydroxyl bridge structures then undergo further hydrolysis, ultimately resulting in the formation of polynuclear hydroxyl bridge complex ions. The interaction between these polynuclear hydroxyl bridge complex ions and the carboxyl groups within the polymer matrix gives rise to the establishment of a network-like gel structure [56,57,58,59].

**Figure 8:**
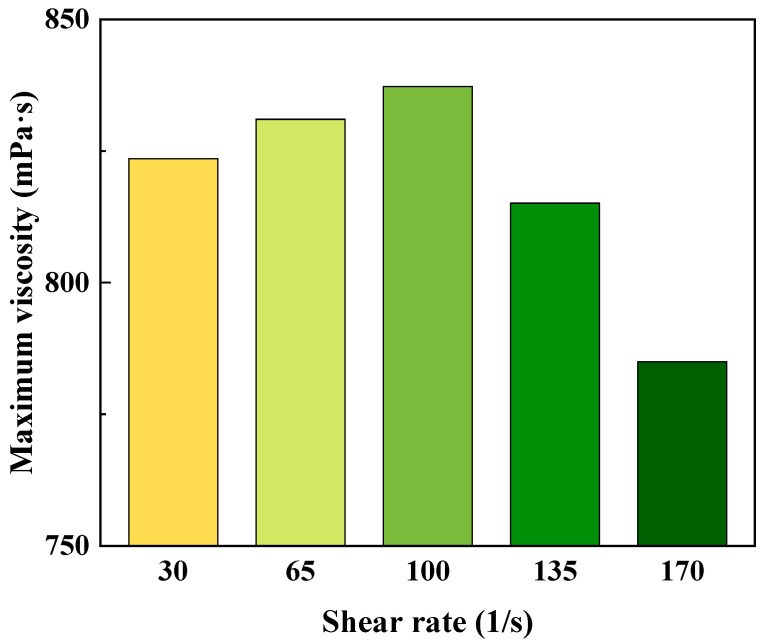
The variation curve of maximum viscosity with respect to shear rate for the polymer gel.

To investigate the kinetic mechanism underlying the crosslinking reaction of the ultra-high-temperature-resistant polymer gel, assessments were conducted to determine the maximum viscosity of the polymer and crosslinking agent mixture during gelation process at varying shear rates and ambient temperature. The outcomes, illustrated in Figure 8, reveal a distinct pattern wherein the maximum viscosity initially increases and subsequently diminishes with escalating shear rates. This trend is primarily attributed to the dual effect of shear rate elevation. On one hand, an augmented shear rate enhances the contact reaction between crosslinking agent and polymer molecules. Conversely, as the shear rate attains higher levels, its disruptive impact on the structural integrity of the polymer gel gains prominence, leading to a decline in the maximum viscosity.

**Figure 9:**
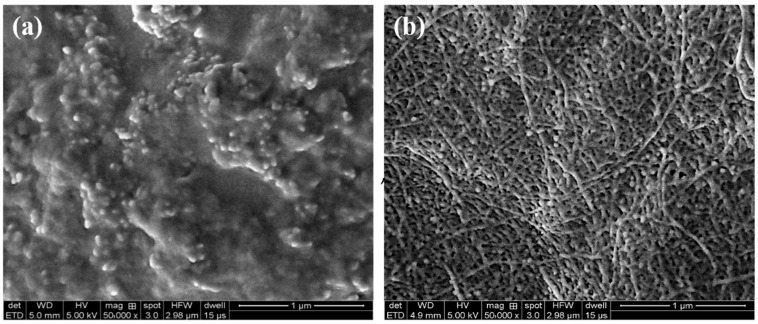
Microstructure of the polymer base liquid (**a**) and polymer gel (**b**).

To gain deeper insights into the gelation mechanism of the polymer gel, scanning electron microscopy (SEM) was employed to scrutinize the microstructure of both the polymer base liquid and the polymer gel. Illustrated in Figure 9, the microstructure of the polymer base liquid unveils a linear bone-like arrangement characterized by closely interconnected branched chains. The intertwined molecular chains configure into a distinctive elevated “fishbone” pattern. Within the solution, the polymer molecules adopt a dispersed state, assuming a random coil configuration. This configuration causes a substantial portion of carboxyl groups within the molecule to be enfolded by these coils, resulting in their diminished participation in the crosslinking reaction. Consequently, the intermolecular crosslinking fails to materialize, leading to minimal modifications in viscosity. Upon the initiation of gelation, the zirconium ions serve as catalysts, inducing the formation of polynuclear hydroxyl bridge complex ions. These complex ions play a pivotal role in linking the surrounding polymer molecular chains, thereby fostering the emergence of a densely packed spatial network structure. In cumulative effect, a considerable number of polymer molecules partake in crosslinking, resulting in a significant elevation in viscosity. Consequently, the polymer gel exhibits heightened stability even when exposed to elevated temperatures. The coherent network structure of the gel efficiently facilitates the transportation of a greater quantity of proppants to effectively support the fractures.

## 3. Conclusions

In this work, a temperature-resistant polymer was synthesized via the solution polymerization method, and an ultra-high-temperature-resistant polymer gel was formulated by introducing an organic zirconium crosslinking agent. The steady shear property, dynamic viscoelasticity, and temperature and shear resistance performance, as well as core damage performance of the polymer gel, were systematically investigated and assessed. The results showcase the exceptional temperature and shear resistance of the polymer gel, with viscosity maintained at 147 mPa·s at a temperature of 200 °C. Impressively, the maximum attainable temperature for the prepared polymer gel displayed an increase, surpassing 20 °C in comparison with analogous gel formulations. When compared with the 30.9% average core damage rate observed in the guanidine gum fracturing fluid, the polymer gel demonstrated a notably diminished average core damage rate, measuring merely 16.6%. Moreover, an in-depth inquiry into the gelation mechanism of the polymer gel was conducted in conjunction with the analysis of microscopic morphology. These findings are poised to be instrumental in underpinning the effective progress of deep and ultradeep oil and gas reservoir development, thereby making a substantive contribution to the sustainable utilization of energy resources.

## 4. Materials and Methods

### 4.1. Materials

In this work, 2-acrylamido-2-methyl-1-propanesulfonic acid (AMPS, 98.0 wt%), acrylic acid (AA, 99.0 wt%), acrylamide (AM, 99.0 wt%), and 1-vinyl-2-pyrrolidone (NVP, 99.0 wt%) were purchased from Shanghai Aladdin Reagent Co., Ltd., Shanghai, China. (NH_4_)_2_S_2_O_8_ (99.9 wt%), and NaHSO_3_ (99.9 wt%) were purchased from Sino-pharm Chemical Reagent Co., Ltd., Shanghai, China. Organic zirconium crosslinking agent was obtained from Shengli Oilfield, Dongying, China. All chemicals were used as received without further purification. Deionized water and simulated formation water (3 wt% NaCl + 0.05 wt% CaCl_2_) were prepared in the lab. The artificial sandstone cores (10 cm in length and 2.5 cm in diameter) were purchased from Haian Oil Scientific Research Instruments Co., Ltd., Nantong, China.

### 4.2. Synthesis of the Temperature-Resistant Polymer

**Figure 10:**
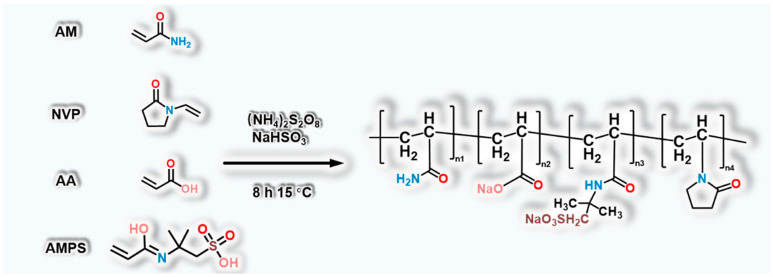
Reaction structure of the temperature-resistant polymer.

In this work, a temperature-resistant polymer was synthesized utilizing the solution polymerization method [60,61], as outlined in Figure 10. Due to its elevated polymerization reactivity and the substantial bonding energy of carbon–carbon bonds, AM was chosen as the primary polymerization monomer. This choice bestows upon the polymer an enhanced temperature resistance and a more pronounced effect in increasing viscosity. To further bolster its temperature and shear resistance, the temperature-resistant monomers, AMPS and NVP, were introduced. Notably, the incorporation of AMPS, with its extensive side group along the chain, imparts superior temperature resistance to the polymer. The presence of sulfonic acid groups augments the solubility of polymer in water, thereby contributing to improved salt resistance. Meanwhile, NVP, characterized by a cyclic structural motif, instigates the formation of rigid side groups that enhance the temperature resistance of polymer. Simultaneously, the introduction of AA introduces carboxyl groups that act as reactive sites for chemical crosslinking with the crosslinking agent. Throughout the experimental process, variations in reaction temperature and duration were introduced. The dissolution duration of the polymerization products and their corresponding viscosities were scrupulously documented. These parameters were subsequently harnessed to refine the optimal experimental conditions.

The synthesis procedure was conducted as follows: Initially, 8.5 g of AMPS was dissolved in 65 g of deionized water, and the pH was adjusted to neutral. Subsequently, 0.4 g of AA, 26 g of AM, and 0.1 g of NVP were sequentially introduced into the solution. The reaction mixture was then placed in a 15 °C water bath, and nitrogen was introduced into the system for 30 min. Following this, 1 g each of the initiators, (NH_4_)_2_S_2_O_8_ and NaHSO_3_, was added. After an 8 h reaction period, the temperature-resistant polymer was successfully synthesized.

### 4.3. Structure Characterization

Thermogravimetric experiments were conducted using the TG 209 F3 Tarsus Thermogravimetric Analyzer (NETZSCH Group, Bavaria, Germany) under a nitrogen atmosphere, encompassing a temperature range from 30 °C to 600 °C, with a temperature ramp rate of 10 °C/min. The designated temperature range delineates distinct stages, each characterized by varying degrees of mass loss within the polymer. Through a comprehensive assessment of the extent of mass loss and comparative analysis of these stages, the thermal stability of the synthesized polymer was systematically probed. Furthermore, the synthesized polymer was subjected to FTIR analysis utilizing the KBr method, employing the Nicolet iS 10 FTIR Spectrometer (Thermo Fisher Scientific, Waltham, MA, USA). This analytical approach yielded valuable insights into the molecular structure and the array of functional groups present in the polymer [62]. To gain a deeper understanding of the gelation mechanism of the polymer gel, SEM was employed with the Sigma 300 Scanning Electron Microscope (ZEISS Group, Guangzhou, China). This facilitated the examination of the microstructure exhibited by both the polymer base liquid and the polymer gel.

### 4.4. Concentration Optimization of Polymer and Crosslinking Agent

Based on preliminary test results, it was observed that a crosslinking agent concentration below 0.6 wt% did not meet the industry standard of retained viscosity exceeding 50 mPa·s. Consequently, the crosslinking agent concentration was temporarily fixed at 0.6 wt%, while variations in polymer concentration were examined to determine the retained viscosity of the gels after subjecting them to 120 min of shear at 200 °C and 170 s^−1^. This approach was adopted to optimize the polymer concentration. Subsequently, the polymer concentration was held constant, and adjustments were made to the crosslinking agent concentration to assess the retained viscosity of the gels under the same experimental conditions, with the aim of optimizing the crosslinking agent concentration.

### 4.5. Rheological Properties Test

The rheological properties of the polymer gel were evaluated using a HAAKE MARS 60 rheometer (Thermo Fisher, Karlsruhe, Germany). The variations in viscosity and shear stress were studied across a shear rate range of 0.1 to 1000 s^−1^, while maintaining a controlled temperature of 25 °C. In order to ascertain the linear viscoelastic range of the polymer gel, stress scans were carried out at a frequency of 1 Hz and a stress level of 1 Pa. These scans provided insights into the viscoelastic behavior of the polymer gel under controlled stress conditions. For assessing the temperature and shear resistance of the polymer gel, tests were conducted at a temperature of 200 °C, a shear rate of 170 s^−1^, and a duration of 120 min. The temperature was gradually increased from 25 °C to 200 °C over a ramping time of 60 min, with a ramping rate of 2.92 °C/min.

### 4.6. Core Damage Test

The prepared polymer gel and conventional guanidine gum fracturing fluid system underwent core damage tests, with the detailed experimental procedures outlined as follows:

(1) A low-permeability sandstone core measuring 10 cm in length was extracted and subsequently divided into two equal-length sections, which were then appropriately labeled. The cut cores underwent ultrasonic vibration cleaning to effectively cleanse the core surface, followed by drying in an oven at 110 °C for a duration of 48 h. Essential parameters, including length, diameter, and permeability of the cores, were meticulously measured and documented. To ensure consistency, simulated formation water was prepared, and the cores were subjected to vacuum saturation with simulated formation water for a span of 24 h. Following this, the polymer gel was broken using 0.1 wt% (NH_4_)_2_S_2_O_8_. Once the gel was completely broken, the lower layer of the gel-breaking liquid was isolated for subsequent use by placing it in a water bath at 100 °C.
(2) The experimental temperature was maintained at 100 °C. The core sample was securely positioned within the core gripper, with the surrounding pressure meticulously adjusted to 3.5 MPa. To introduce simulated formation water into the core, a fixed-flow pump in the form of an advection plunger pump was employed, operating at a consistent flow rate of 0.1 mL/min. To ensure reliable results, the system was allowed to reach a stable state for a minimum duration of 60 min, during which the pressure difference was carefully monitored and recorded.
(3) The core gripper was carefully opened, and the core was rotated back and forth before being placed back into the core gripper. A consistent surrounding pressure of 3.5 MPa was meticulously applied, and the plunger pump was engaged to administer the gel-breaking liquid into the core at a precisely controlled flow rate of 1 mL/min. This injection process was maintained for a duration of 36 min. Subsequent to this, the valves situated at both ends of the gripper were securely closed, and a waiting period of 2 h was meticulously observed.
(4) Subsequently, the core gripper was reopened, and the core was once again rotated back and forth before being placed back into the core gripper. The previously delineated procedures were systematically replicated, and the resultant stable pressure difference was meticulously logged. The assessment of core permeability, both prior to and post the inflicted damage, was quantitatively determined utilizing Darcy’s formula:

(1) $$k=10^{-1}\frac{Q\mu L}{A\Delta p}$$

where k is permeability in mD, μ represents the viscosity of simulate formation water in mPa·s, L is the core length in cm, Q is the flow rate through the core in mL/s, Δp represents the core inlet and outlet pressure difference in 10^−3^ MPa, and A denotes the core cross-sectional area in cm^2^.

The core damage rate can be computed as

(2) $$\eta_{d}=\frac{K_{0}-K_{1}}{K_{0}}\times 100\%$$

where $\eta_{d}$ is the core damage rate in %, $K_{0}$ represents the matrix permeability before gel-breaking liquid entry in mD, and $K_{1}$ denotes the matrix permeability upon gel-breaking liquid entry in mD.

## Figures and Tables

**Table 1:** Specific core parameters and experimental conditions.

System	Number	Length/cm	Diameter/cm	Initial Permeability/mD	Damage Permeability/mD	Damage Rate/%	Average Damage Rate/%	RSD/%
polymer gel	1	5.01	2.50	2.30	1.93	16.1	16.6	3.03
	2	4.98	2.50	2.18	1.82	16.5		
	3	4.98	2.50	2.34	1.94	17.1		
guanidine gum	1	4.99	2.50	2.26	1.57	30.5	30.9	4.49
	2	5.02	2.50	2.53	1.71	32.4		
	3	4.95	2.50	2.59	1.82	29.7		

## Data Availability

Not applicable.

## References

[1] Yang D., Peng X., Peng Q., Wang T., Qiao C., Zhao Z., Gong L., Liu Y., Zhang H., Zeng H. (2022). Probing the Interfacial Forces and Surface Interaction Mechanisms in Petroleum Production Processes. Engineering.

[2] Zhao M., Liu S., Dai C., Yan R., Li Y., Liu P. (2023). Development and Drag Reduction Behaviors of a Water-in-Water Emulsion Polymer Drag Reducer. ACS Appl. Polym. Mater..

[3] Guo X., Hu D., Li Y., Duan J., Zhang X., Fan X., Duan H., Li W. (2019). Theoretical Progress and Key Technologies of Onshore Ultra-Deep Oil/Gas Exploration. Engineering.

[4] Hu D., Ren L., Li Z., Zhao J., Lin R., Jiang T. (2022). Simulation of fracture control during temporary plugging at fracture openings in deep and ultra-deep shale-gas horizontal wells. Nat. Gas Ind. B.

[5] Lei Q., Xu Y., Yang Z., Cai B., Wang X., Zhou L., Liu H., Xu M., Wang L., Li S. (2021). Progress and development directions of stimulation techniques for ultra-deep oil and gas reservoirs. Pet. Explor. Dev..

[6] Li J., Wang Y., Ma W., Wang D., Ma C., Li Z. (2015). Evaluation on occluded hydrocarbon in deep–ultra deep ancient source rocks and its cracked gas resources. Nat. Gas Ind. B.

[7] Wu X., Li X., Ping J., Ying Y. (2021). Recent advances in water-driven triboelectric nanogenerators based on hydrophobic interfaces. Nano Energy.

[8] Liu H., Wang F., Zhang J., Meng S., Duan Y. (2014). Fracturing with carbon dioxide: Application status and development trend. Pet. Explor. Dev..

[9] Osiptsov A.A. (2017). Fluid Mechanics of Hydraulic Fracturing: A Review. J. Pet. Sci. Eng..

[10] Huang Q., Liu S., Cheng W., Wang G. (2020). Fracture permeability damage and recovery behaviors with fracturing fluid treatment of coal: An experimental study. Fuel.

[11] Wang J., Elsworth D., Wu Y., Liu J., Zhu W., Liu Y. (2018). The Influence of Fracturing Fluids on Fracturing Processes: A Comparison Between Water, Oil and SC-CO_2_. Rock Mech. Rock Eng..

[12] Wang J., Wang S., Lin W., Kang Z., You Q. (2017). Formula optimization and rheology study of clean fracturing fluid. J. Mol. Liq..

[13] Yang B., Zhao J., Mao J., Tan H., Zhang Y., Song Z. (2019). Review of friction reducers used in slickwater fracturing fluids for shale gas reservoirs. J. Nat. Gas Sci. Eng..

[14] Wang X., Zhao M., Wang X., Liu P., Fan M., Yan X., Ma Z., Zhang Y., Dai C. (2023). Synergistic effect of dual hydrogen-donor deep eutectic solvent for performance improvement of fracturing-oil expulsion fluids. Chem. Eng. J..

[15] Xie K., Mei J., Cao W., Cao B., Yao L., Zhang B., Wang H., Guo K., Wu Z., Yan K. (2022). Improving oil mechanism of polymer gel fracturing fluid based on filtration displacement. J. Pet. Sci. Eng..

[16] Wang J., Zhou F., Bai H., Li Y., Yang H. (2020). A Comprehensive method to evaluate the viscous slickwater as fracturing fluids for hydraulic fracturing applications. J. Pet. Sci. Eng..

[17] Liu P., Dai C., Gao M., Wang X., Liu S., Jin X., Li T., Zhao M. (2022). Development of the Gemini Gel-Forming Surfactant with Ultra-High Temperature Resistance to 200 °C. Gels.

[18] Esmaeilirad N., White S., Terry C., Prior A., Carlson K. (2016). Influence of inorganic ions in recycled produced water on gel-based hydraulic fracturing fluid viscosity. J. Pet. Sci. Eng..

[19] Wang M., Wu W., Chen S., Li S., Li T., Ni G., Fu Y., Zhou W. (2022). Experimental Evaluation of the Rheological Properties and Influencing Factors of Gel Fracturing Fluid Mixed with CO_2_ for Shale Gas Reservoir Stimulation. Gels.

[20] Hayashi S.-i., Fujiwara F., Usui S., Tominaga T. (2012). Effect of inorganic salt on the dose sensitivity of polymer gel dosimeter. Radiat. Phys. Chem..

[21] Zaharaki D., Komnitsas K., Perdikatsis V. (2010). Use of analytical techniques for identification of inorganic polymer gel composition. J. Mater. Sci..

[22] Zhu D., Bai B., Hou J. (2017). Polymer Gel Systems for Water Management in High-Temperature Petroleum Reservoirs: A Chemical Review. Energy Fuels.

[23] Khan F., Tandon A., Dhar V., Beg M., Saxena A., Sharma S. (2022). Cement Slurry Design for High-Pressure, High-Temperature Wellbores Using Polymer Nanocomposite: A Laboratory Scale Study Based on the Compressive Strength and Fluid Loss. Energy Fuels.

[24] Dastan S., Hassnajili S., Abdollahi E. (2016). Hydrophobically associating terpolymers of acrylamide, alkyl acrylamide, and methacrylic acid as EOR thickeners. J. Polym. Res..

[25] Ghaderi S., Ramazani S.A.A., Haddadi S.A. (2019). Applications of highly salt and highly temperature resistance terpolymer of acrylamide/styrene/maleic anhydride monomers as a rheological modifier: Rheological and corrosion protection properties studies. J. Mol. Liq..

[26] Van Mastrigt F., Stoffelsma T., Wever D.A.Z., Picchioni F. (2017). Thermoresponsive comb polymers as thickeners for high temperature aqueous fluids. Mater. Today Commun..

[27] Zheng C., Hou Z., Xu K., Weng D., Hou Z., Shi Y., Lai J., Liu C., Wang T. (2023). Preparation and rheological properties of acrylamide-based penta-polymer for ultra-high temperature fracturing fluid. Colloids Surf. A Physicochem. Eng. Asp..

[28] Mao J., Xue J., Zhang H., Yang X., Lin C., Wang Q., Li C., Liao Z. (2022). Investigation of a hydrophobically associating polymer’s temperature and salt resistance for fracturing fluid thickener. Colloid Polym. Sci..

[29] Du J., Liu J., Zhao L., Liu P., Chen X., Wang Q., Yu M. (2022). Water-soluble polymers for high-temperature resistant hydraulic fracturing: A review. J. Nat. Gas Sci. Eng..

[30] He Y., Gou S., Zhou Y., Zhou L., Tang L., Liu L., Fang S. (2020). Thermoresponsive behaviors of novel polyoxyethylene-functionalized acrylamide copolymers: Water solubility, rheological properties and surface activity. J. Mol. Liq..

[31] Nesrinne S., Djamel A. (2017). Synthesis, characterization and rheological behavior of pH sensitive poly(acrylamide-co-acrylic acid) hydrogels. Arab. J. Chem..

[32] Liu Y.-L., Su Y.-H., Lai J.-Y. (2004). In situ crosslinking of chitosan and formation of chitosan–silica hybrid membranes with using γ-glycidoxypropyltrimethoxysilane as a crosslinking agent. Polymer.

[33] Wong R.S., Ashton M., Dodou K. (2015). Effect of Crosslinking Agent Concentration on the Properties of Unmedicated Hydrogels. Pharmaceutics.

[34] Nianyin L., Yu J., Daocheng W., Chao W., Jia K., Pingli L., Chengzhi H., Ying X. (2022). Development status of crosslinking agent in high-temperature and pressure fracturing fluid: A review. J. Nat. Gas Sci. Eng..

[35] Zhao M., Li Y., Xu Z., Wang K., Gao M., Lv W., Dai C. (2020). Dynamic cross-linking mechanism of acid gel fracturing fluid. Colloids Surf. A Physicochem. Eng. Asp..

[36] Kamal M.S., Mohammed M., Mahmoud M., Elkatatny S. (2018). Development of Chelating Agent-Based Polymeric Gel System for Hydraulic Fracturing. Energies.

[37] Liu Q., Yu L., Wang Y., Ji Y., Horvat J., Cheng M.-L., Jia X., Wang G. (2013). Manganese-Based Layered Coordination Polymer: Synthesis, Structural Characterization, Magnetic Property, and Electrochemical Performance in Lithium-Ion Batteries. Inorg. Chem..

[38] Puts G.J., Crouse P., Ameduri B.M. (2019). Polytetrafluoroethylene: Synthesis and Characterization of the Original Extreme Polymer. Chem. Rev..

[39] Shawky H.A., Chae S.-R., Lin S., Wiesner M.R. (2011). Synthesis and characterization of a carbon nanotube/polymer nanocomposite membrane for water treatment. Desalination.

[40] Bach Q.-V., Chen W.-H. (2017). Pyrolysis characteristics and kinetics of microalgae via thermogravimetric analysis (TGA): A state-of-the-art review. Bioresour. Technol..

[41] Carrier M., Loppinet-Serani A., Denux D., Lasnier J.-M., Ham-Pichavant F., Cansell F., Aymonier C. (2011). Thermogravimetric analysis as a new method to determine the lignocellulosic composition of biomass. Biomass Bioenergy.

[42] Loganathan S., Valapa R.B., Mishra R.K., Pugazhenthi G., Thomas S., Thomas S., Thomas R., Zachariah A.K., Mishra R.K. (2017). Chapter 4—Thermogravimetric Analysis for Characterization of Nanomaterials. Thermal and Rheological Measurement Techniques for Nanomaterials Characterization.

[43] Bair S., Yamaguchi T., Brouwer L., Schwarze H., Vergne P., Poll G. (2014). Oscillatory and steady shear viscosity: The Cox–Merz rule, superposition, and application to EHL friction. Tribol. Int..

[44] Jasso M., Polacco G., Zanzotto L. (2022). Shear Viscosity Overshoots in Polymer Modified Asphalts. Materials.

[45] Liu S., Zhao M., Wu Y., Gao Z., Dai C., Liu P. (2022). Development and Performance Evaluation of a Novel Silica Nanoparticle-Reinforced CO_2_-Sensitive Fracturing Fluid with High Temperature and Shear Resistance Ability. Energy Fuels.

[46] Yang H., Shao S., Zhu T., Chen C., Liu S., Zhou B., Hou X., Zhang Y., Kang W. (2019). Shear resistance performance of low elastic polymer microspheres used for conformance control treatment. J. Ind. Eng. Chem..

[47] Yang J., Bai Y., Sun J., Lv K., Han J., Dai L. (2022). Experimental Study on Physicochemical Properties of a Shear Thixotropic Polymer Gel for Lost Circulation Control. Gels.

[48] Zhang C., Wang Y., Wang Z., Wang H., Liang S., Xu N., Li D. (2023). Mechanism Analysis of Enhancing the Temperature and Shear Resistance of Hydroxypropyl Guar Gum Fracturing Fluid by Boron-Functionalized Nanosilica Colloidal Crosslinker. Colloids Surf. A Physicochem. Eng. Asp..

[49] Yu Y., Wang H., Wang Y.-n., Zhou J., Shi B. (2022). Chrome-free synergistic tanning system based on biomass-derived hydroxycarboxylic acid–zirconium complexes. J. Clean. Prod..

[50] Fu L., Liao K., Ge J., Huang W., Chen L., Sun X., Zhang S. (2020). Study on the damage and control method of fracturing fluid to tight reservoir matrix. J. Nat. Gas Sci. Eng..

[51] Lufeng Z., Fujian Z., Shicheng Z., Zhun L., Jin W., Yuechun W. (2019). Evaluation of permeability damage caused by drilling and fracturing fluids in tight low permeability sandstone reservoirs. J. Pet. Sci. Eng..

[52] Tang H., Tang H., He J., Zhao F., Zhang L., Liao J., Wang Q., Yuan X. (2021). Damage mechanism of water-based fracturing fluid to tight sandstone gas reservoirs: Improvement of The Evaluation Measurement for Properties of Water-based Fracturing Fluid: SY/T 5107-2016. Nat. Gas Ind. B.

[53] Fan Y., Duan W., Xu K., Yan C., Zheng C. (2023). Zr, N-Co-Doped Carbon Quantum Dot Crosslinking Agents for Use in Fracturing Fluids. ACS Appl. Nano Mater..

[54] Milošev I., Kapun B., Rodič P., Iskra J. (2015). Hybrid sol–gel coating agents based on zirconium(IV) propoxide and epoxysilane. J. Sol-Gel Sci. Technol..

[55] Zhou M., Zhang J., Zuo Z., Liao M., Peng’ao P. (2021). Preparation and property evaluation of a temperature-resistant Zr-crosslinked fracturing fluid. J. Ind. Eng. Chem..

[56] Jiang H., Zhang G., Feng X., Liu H., Li F., Wang M., Li H. (2017). Room-temperature self-healing tough nanocomposite hydrogel crosslinked by zirconium hydroxide nanoparticles. Compos. Sci. Technol..

[57] Ma L., Xie J., Yan X., Fan Z., Li H., Lu L., Chen L., Xin Y., Yin P. (2022). Wearable membranes from zirconium-oxo clusters cross-linked polymer networks for ultrafast chemical warfare agents decontamination. Chin. Chem. Lett..

[58] Othman A., Aljawad M.S., Kamal M.S., Mahmoud M., Patil S., Alkhowaildi M. (2022). Rheological Study of Seawater-Based Fracturing Fluid Containing Polymer, Crosslinker, and Chelating Agent. ACS Omega.

[59] Othman A., Aljawad M.S., Kamal M.S., Mahmoud M., Patil S., Kalgaonkar R. (2023). Individual Seawater Ions’ Impact on the Rheology of Crosslinked Polymers in the Presence of a Chelating Agent. Energy Fuels.

[60] Abdollahi Z., Frounchi M., Dadbin S. (2011). Synthesis, characterization and comparison of PAM, cationic PDMC and P(AM-co-DMC) based on solution polymerization. J. Ind. Eng. Chem..

[61] Lin J., Wu X., Zheng C., Zhang P., Huang B., Guo N., Jin L. (2014). Synthesis and properties of epoxy-polyurethane/silica nanocomposites by a novel sol method and in-situ solution polymerization route. Appl. Surf. Sci..

[62] Ramesh S., Leen K.H., Kumutha K., Arof A.K. (2007). FTIR studies of PVC/PMMA blend based polymer electrolytes. Spectrochim. Acta Part A Mol. Biomol. Spectrosc..

